# Supplementary material for: Quality of Life as a Mediator between Cancer Stage and Long-Term Mortality in Nasopharyngeal Cancer Patients Treated with Intensity-Modulated Radiotherapy
Source: Cancers (Basel). 2021 Oct 10;13(20):5063. doi: 10.3390/cancers13205063 (PMC8533735; doi:10.3390/cancers13205063)
Supplement: Supplementary file 1 [file cancers-13-05063-s001.zip › cancers-1346845-supplementary.pdf]

Supplementary Materials

Quality of Life as a Mediator between Cancer Stage and Long Term Mortality in Nasopharyngeal Cancer Patients Treated with Intensity-Modulated Radiotherapy

Kuan-Cho Liao, Hui-Ching Chuang, Chih-Yen Chien, Yu-Tsai Lin, Ming-Hsien Tsai, Yan-Ye Su, Chao-Hui Yang, Chi-Chih Lai, Tai-Lin Huang, Shau-Hsuan Li, Tsair-Fwu Lee, Wei-Ting Lin, Chien-Hung Lee and Fu-Min Fang

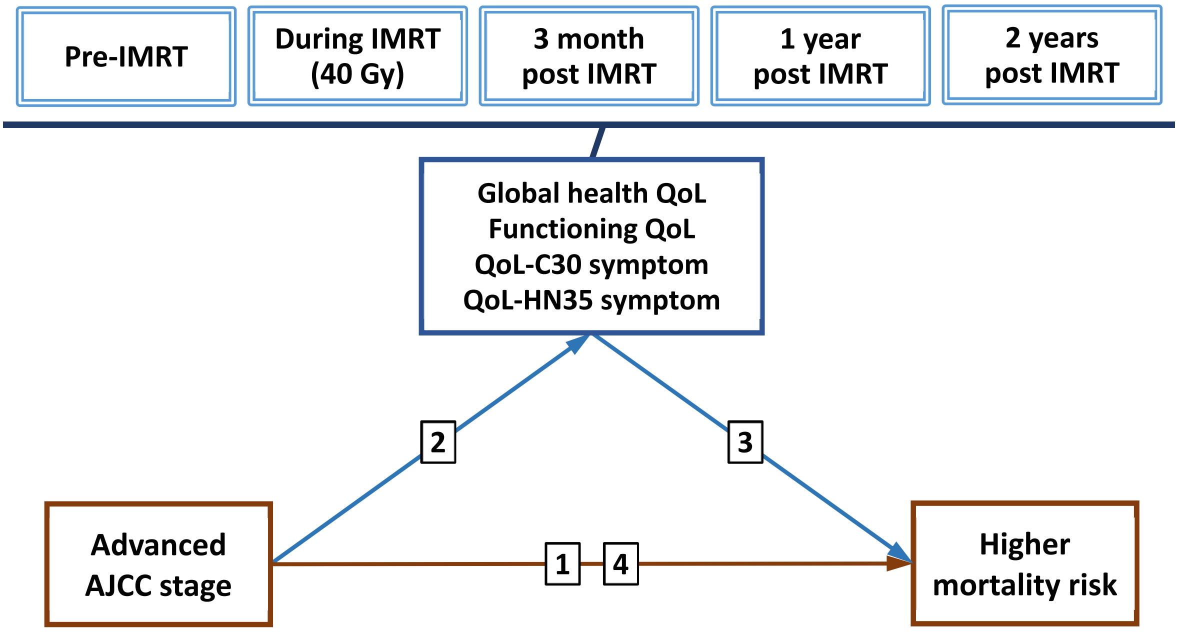

Figure S1. The processes of assessing quality of life (QoL) at different intensity-modulated radiation therapy (IMRT)-related time periods as a possible mediator on the relationship between advanced AJCC tumor stage (III-IV) and higher mortality risk in nasopharyngeal cancer patient follow-up cohort. If the paths 1, 2 and 3 exist at the same time, it denotes possible intermediated effects. Verification procedure: (1) the association between advanced stage and mortality risk, (2) the association between advanced stage and specific QoL scales, (3) the association between specific QoL scales after controlling for advanced stage, and (4) the association between advanced stage and mortality risk after adjusting for a specific QoL-mediator. Note: Global health QoL, functioning QoL, QoL-C30 symptom, and QoL-HN35 symptom denoted the average QoL scores obtained from the EORTC QLQ-C30 global QoL scale, 5 functional scales, and 9 symptom scales/items, and EORTC QLQ-HN35 18 symptom scales/items, respectively. AJCC, American Joint Committee on Cancer staging system; EORTC, European organization for research and treatment of cancer; QoL-C30, the core QoL questionnaire of EORTC; QoL-HN35, the head and neck cancer-specific QoL questionnaire module of EORTC.

Table S1. Distributions of quality of life (QoL) scores of EORTC QLQ-C30 and EORTC QLQ-HN35 at different IMRT-related time points for nasopharyngeal cancer patients.

Quality of Life Score [a]	Pretreatment of IMRT	IMRT Period	After IMRT		
			3 months	1 year	2 years
EORTC QLQ-C30 [b]					
**Global health QoL**	**53.1±20.9**	**39.9±19.0**	**57.1±19.3**	**63.8±19.1**	**65.3±18.8**
**Functioning QoL**	**84.0±13.4**	**78.0±15.3**	**83.2±13.2**	**85.8±13.1**	**87.0±11.7**
Physical functioning	92.3±12.6	84.7±14.8	87.2±13.3	89.8±13.0	90.3±11.4
Role functioning	90.8±20.8	79.6±24.5	86.9±18.8	91.6±16.3	92.9±14.7
Emotional functioning	76.1±19.2	76.0±18.7	81.9±17.4	83.5±17.3	84.1±16.5
Cognitive functioning	85.3±17.0	81.2±18.1	83.7±17.2	83.2±17.5	82.7±17.7
Social functioning	75.4±22.7	68.5±23.0	76.2±20.4	81.0±20.3	85.1±17.6
**QoL-C30 symptom**	**16.3±12.9**	**28.4±13.6**	**18.5±12.8**	**14.7±12.4**	**13.5±10.9**
Fatigue	22.7±19.4	38.8±19.4	29.9±18.1	24.8±18.2	24.1±17.6
Nausea and vomiting	8.1±16.1	32.0±24.4	10.5±17.0	4.8±10.9	4.1±10.3
Pain	16.5±20.9	31.2±23.4	16.5±18.9	13.5±17.8	12.2±15.4
Dyspnea	8.8±16.0	11.7±18.7	9.4±16.5	7.9±15.7	10.0±16.1
Insomnia	26.2±25.9	28.1±24.8	23.7±25.2	22.8±24.1	20.7±23.0
Appetite loss	15.7±22.8	51.1±27.2	25.6±24.2	13.7±20.1	9.9±16.5
Constipation	11.6±19.0	23.5±23.5	17.0±21.4	14.1±20.3	13.1±19.7
Diarrhea	11.7±17.7	14.8±20.0	11.2±17.2	9.3±15.7	9.2±16.0
Financial difficulties	25.6±26.3	24.8±25.8	22.9±24.6	21.3±25.2	18.0±23.8
EORTC QLQ-HN35 [b]					
**QoL-HN35 symptom**	**16.0±11.4**	**38.4±14.3**	**27.1±13.0**	**22.4±13.0**	**19.8±12.2**
Pain	8.5±13.1	35.3±22.4	17.2±17.2	10.3±14.7	9.4±13.6
Swallowing	6.9±12.4	36.5±23.4	20.0±17.9	16.2±17.5	14.5±15.9
Senses problems	8.7±16.5	44.7±23.3	26.0±22.5	19.0±21.1	16.9±20.2
Speech problems	8.3±13.0	22.8±21.9	16.7±17.4	12.9±15.4	10.9±14.6
Trouble with social eating	6.6±13.4	41.9±25.6	22.2±20.3	13.9±18.3	11.5±16.1
Trouble with social contact	5.3±11.2	18.9±19.8	11.8±15.3	8.1±13.3	6.4±11.5
Less sexuality	15.1±22.1	34.7±30.1	26.6±24.7	22.5±24.6	20.0±22.8
Teeth	24.0±23.8	22.6±23.5	25.8±24.1	24.6±23.1	24.1±23.8
Opening mouth	6.4±15.0	22.3±24.6	15.9±21.3	14.1±20.1	13.5±19.1
Dry mouth	23.1±23.3	57.3±25.9	56.1±25.9	47.9±26.1	41.8±25.2
Sticky saliva	15.2±21.1	54.2±28.2	44.4±28.3	35.2±26.9	30.4±25.0
Coughing	21.4±20.9	33.6±24.9	21.9±20.3	20.2±20.9	20.2±19.8
Felt ill	22.3±22.8	44.2±27.3	26.7±22.0	20.2±20.3	18.9±18.8
Painkillers	35.4±47.6	50.5±49.4	18.8±38.8	15.9±36.3	16.3±36.8
Nutritional supplements	31.9±46.4	73.9±43.4	50.3±50.0	39.2±48.6	34.0±47.2
Feeding tube	1.4±11.4	5.6±22.8	3.9±19.3	3.7±18.8	1.8±13.3
Weight loss	32.9±46.6	77.2±41.3	51.9±49.7	27.3±44.3	19.2±39.2
Weight gain	15.3±35.4	15.2±34.8	31.3±45.9	53.0±49.5	47.5±49.8

[a] Data are expressed in Mean ± Standard Deviation; [b] Global health QoL, functioning QoL, QoL-C30 symptom, and QoL-HN35 symptom denoted the average QoL scores obtained from the EORTC QLQ-C30 global QoL scale, 5 functional scales, and 9 symptom scales/items, and EORTC QLQ-HN35 18 symptom scales/items, respectively. EORTC, European organization for research and treatment of cancer; QoL-C30, the core QoL questionnaire of EORTC; QoL-HN35, the head and neck cancer-specific QoL questionnaire module of EORTC.

© 2021 by the authors. Submitted for possible open access publication under the terms and conditions of the Creative Commons Attribution (CC BY) license (http://creativecommons.org/licenses/by/4.0/).
